# Supervised smoking facility access, harm reduction practices, and substance use changes during the COVID-19 pandemic: a community-engaged cross-sectional study

**DOI:** 10.1186/s12954-023-00825-7

**Published:** 2023-07-31

**Authors:** Jenna van Draanen, Jonah Hamilton, Jeffrey Morgan, Scott Maxwell, Tara Taylor, Lindsey Richardson, Seonaid Nolan

**Affiliations:** 1 BC Centre On Substance Use, 400 - 1045 Howe Street, Vancouver, BC V6Z 2A9 Canada; 2 Child, Family, and Population Health Nursing, Health Sciences Building, University of Washington, Box 357262, Seattle, WA 98195-0005 USA; 3 School of Population and Public Health, University of British Columbia, 2206 East Mall, Vancouver, BC V6T 1Z3 Canada; 4 Overdose Prevention Participatory Research Assistant Program, Overdose Prevention Society, 58 E Hastings St, Vancouver, BC V6A 1N1 Canada; 5 Spencer Creo Foundation, 500-610 Main St, Vancouver, BC V6A 2V3 Canada; 6 Department of Sociology, Faculty of Arts, University of British Columbia, 6303 NW Marine Drive, Vancouver, BC V6T 1Z1 Canada; 7 Department of Medicine, Faculty of Medicine, University of British Columbia, 2775 Laurel Street, Vancouver, BC V5Z 1M9 Canada

**Keywords:** Supervised inhalation site, Supervised consumption site, Overdose prevention, Poisoning, Harm reduction

## Abstract

**Background:**

The potential public health benefits of supervised smoking facilities (SSFs) are considerable, and yet implementation of SSFs in North America has been slow. We conducted this study to respond to significant knowledge gaps surrounding SSF utilization and to characterize substance use, harm reduction practices, and service utilization following the onset of the COVID-19 pandemic.

**Methods:**

A questionnaire was self-administered at a single site by 175 clients using an outdoor SSF in Vancouver, Canada, between October–December 2020. Questionnaire responses were summarized using descriptive statistics. Multinomial logistic regression techniques were used to examine factors associated with increased SSF utilization.

**Results:**

Almost all respondents reported daily substance use (93% daily use of opioids; 74% stimulants). Most used opioids (85%) and/or methamphetamine (66%) on the day of their visit to the SSF. Respondents reported drug use practice changes at the onset of COVID-19 to reduce harm, including using supervised consumption sites, not sharing equipment, accessing medically prescribed alternatives, cleaning supplies and surfaces, and stocking up on harm reduction supplies. Importantly, 45% of SSF clients reported using the SSF more often since the start of COVID-19 with 65.2% reporting daily use of the site. Increased substance use was associated with increased use of the SSF, after controlling for covariates.

**Conclusions:**

Clients of the SSF reported increasing not only their substance use, but also their SSF utilization and harm reduction practices following the onset of COVID-19. Increased scope and scale of SSF services to meet these needs are necessary.

**Supplementary Information:**

The online version contains supplementary material available at 10.1186/s12954-023-00825-7.

## Background

Supervised consumption facilities provide a safe and hygienic environment where clients can consume illicit substances under the supervision of trained staff [1]. Supervised consumption facilities have played a critical role in improving access to addiction treatment programs and addressing many injection-related harms and risks [2–4]. Notably, such facilities have reduced injection-related skin infections, syringe sharing, and overdose morbidity and mortality [3] which is particularly relevant in the context of the ongoing and unprecedented drug poisoning and overdose crisis in North America. In addition, supervised consumption facilities also help connect people to health and social services, reduce improper disposal of syringes, and bring other public safety benefits to the neighbourhoods in which they are located [3].

Worldwide, just over 10% of people who consume opioids, amphetamines, and cocaine consume drugs through injection [5]. Most current consumption facilities, especially those outside of Europe [6], operate primarily as supervised injection facilities and do not provide supervised spaces for people who inhale or smoke drugs. This leaves a large service gap with dire consequences, as smoking is increasingly associated with overdose across North America and was, for example, the most common mode of consumption among decedents of drug toxicity deaths in British Columbia, Canada between 2017 and 2022 [7]. As with supervised injection facilities, supervised smoking facilities (SSFs) address several health issues, including reducing drug poisoning fatality, limiting transmission of infectious disease through reduced equipment sharing, and increasing access to health and social services for people who use drugs (PWUD) [8–11]. In addition, SSFs have a documented ability to meet the needs of highly vulnerable people who inhale drugs and can decrease exposure to interpersonal violence [9].

While overall demand for SSF services in North America is underexplored, initial studies in localized contexts identify significant desire to use an SSF among people who consume stimulants [9, 12–14]. Despite the clear potential SSFs have to save lives and reduce harms, current service provision in North America is wholly inadequate to meet this demand: there is only one indoor SSF [8] and two outdoor SSFs in Canada [15]. In one of the few supervised consumption spaces in Canada that includes an SSF, one-third of visits to the space are specifically for the use of smoking facilities [15]. There are longstanding calls for implementation of such spaces because of their established capacity to reduce harm for PWUD [8–11, 15], and activists have periodically provided “pop-up” SSF services for PWUD to meet these critical service gaps [16, 17]. Yet, the expansion of SSF services has not kept pace, and the knowledge required to support successful service implementation (e.g. factors affecting service utilization) has been missing. Specifically, the barriers and facilitators to SSF implementation are not well-known as scientific literature on SSF planning, implementation, operation, utilization, and evaluation is scant [6, 18]. Further, the few SSF sites currently in operation in North America are under-resourced, and often have not had the capacity to engage in research to advance knowledge about the life-saving services they deliver, despite calls for evaluation of these services [18]. This knowledge could improve service delivery and increase implementation of SSFs elsewhere, helping to meet the needs of PWUD who cannot use other supervised consumption facilities due to their preferred route of administration [9].

The implementation of SSFs was further disrupted by COVID-19, which forced existing supervised consumption facilities to adapt operations to protect staff and clients [19–21]. As with many services, public health restrictions that reduced capacity and, in some cases, closed supervised consumption facilities had clear impacts on service delivery and access [20]. This decrease in service capacity tragically occurred alongside increases in risk associated with substance use and overdose events [21, 23, 24]. In response to service disruptions and a variety of social changes associated with COVID-19, some PWUD may have also changed harm reduction strategies to limit their exposure to COVID-19 or drug-related harm [25, 26], yet literature on these changes is limited. The need for knowledge about utilization of SSFs pre-existed the COVID-19 pandemic [18] and has only deepened since with the rapidly changing drug use and harm reduction landscape. This information is critically needed to support both existing and new sites in addressing this important gap in harm reduction services for PWUD during and beyond the global pandemic.

We designed an exploratory study to respond to these knowledge gaps. Using community-engaged methods we collected data from current SSF users at a high-traffic peer-led SSF in Vancouver’s Downtown Eastside neighbourhood, in British Columbia, Canada. At the time of data collection, the organization ran one indoor site where clients could inject drugs (i.e. “injection room”) and one outdoor site where clients could smoke drugs (i.e. “inhalation site” or SSF) in the DTES, three blocks apart. At the time, the outdoor inhalation site had 12 individual booths (each with a separate desk, canopy, chair) staffed by a minimum of five peer workers. The outdoor SSF also had a washroom trailer, monitored by two peer workers who worked as washroom monitors and neighbourhood ambassadors. In addition to the supervised injection and smoking facilities, the organization provided drug testing, harm reduction supplies, jobs and employment opportunities, housing supports, and food distribution.

The organization has been in operation since 2016, and between its multiple sites, at the time of data collection, it averaged approximately 600 visits per day. Being peer-run, their sites are staffed by people with lived and living experience of drug use, a different model than the majority of medically staffed SSF sites globally. In contrast to some of the medically modelled SSF sites globally, the SSF under study operates using a community model, offering economic and employment opportunities that contribute to stability and wellness for the SSF community members. The site is also a uniquely low-barrier SSF, strongly avoiding the use of disciplinary practices such as “bars” or “bans”, which prohibit clients from accessing a site either for a designated time period (e.g. 24 h) or even indefinitely.

During the COVID 19 pandemic, the SSF was required to follow provincial health guidelines to respond to overdose (e.g. avoid using oxygen). They also took additional safeguards to prevent the spread of COVID-19 including giving more space between each booth, conducting extra sanitation practices, and limiting communal drug use on site. The site was not closed during the COVID-19 pandemic, bathroom access and social programs continued, and the site did not reduce their operating hours.

To guide the implementation of safe inhalation services in new contexts and settings, we studied factors that are critical to guiding the implementation of safe inhalation services in new contexts and settings, specifically, barriers and facilitators of accessing the current SSF’s services, program participant characteristics, and participant suggestions for future peer led SSFs. To our knowledge, this is the first implementation-focused study at an SSF. As such, the current study provides insight into service utilization trends which can support current and future SSF planning and operations.

## Methods

### Data collection

A cross-sectional, self-administered questionnaire (see Additional file 1. Appendix A for a copy of the questionnaire) was completed by SSF clients between October and December 2020 to collect demographic, utilization, and service delivery satisfaction data. Data collection windows lasted 2–3 h each and were intentionally scheduled during different times of day, different days of the week, and different weeks of the month to solicit responses from a range of SSF clients.

The SSF site operates as an outdoor facility with separate tents and tables set up for each SSF user in an outdoor lot. Data collection was conducted by two members of the research team, one peer researcher and one formally trained researcher. All SSF clients who entered the site during periods of data collection were invited to participate and were offered $5 in exchange for their participation. Clients were not required to participate in the study. Verbal consent was obtained using a script approved by the Research Ethics Board and the questionnaire included written text saying that submitting the questionnaire meant respondents were consenting to participate in the study.

Cover sheets with information about the study and the informed consent process were given to clients as they entered the site along with questionnaires. If interested in participating in the study, clients took a questionnaire and pencil to their outdoor table/tent and completed the survey in their own private space within the outdoor site. Questionnaires were completed anonymously for participant comfort and safety. Researchers and peer research assistants offered to read and record responses for participants who had literacy barriers. Questionnaires were designed to be completed within 5 min. This study was reviewed and approved by the University of British Columbia/Providence Health Care Research Ethics Board.

### Variables

*Sociodemographic characteristics.* Gender identity (man, woman, non-binary, other), age (< 19, 20–29, 30–39, 40–49, 50–59, 60+), and housing status (where participant slept most nights in past month: street, own house or apartment, with friends/family, shelter, tent, hotel, or other) were collected to record participant demographics. Housing status was further collapsed into a binary homelessness variable (yes/no) where “own house/apartment” was coded “no”, and all other categories were coded “yes”. Given data collection constraints early in the COVID-19 pandemic and privacy considerations for our highly vulnerable and visible study population, we intentionally collected limited demographic information (specifically no race/ethnicity data) from respondents.

*Substance use behaviours.* Substances used at the SSF on the day of the visit included options for: heroin/fentanyl, crystal methamphetamine, cannabis, alcohol, crack, cocaine, other, or not using substances (e.g. using the bathrooms, socializing). Multiple response options were allowed. Substance use frequency variables for broad categories of stimulant use (at least daily/ less than daily stimulant use) and opioid use (at least daily/ less than daily opioid use) were created from substance use frequency questions with response options of: more than once per day, every day, more than once per week, once per week, less than once per week, and never.

*Barriers to access.* Participants were first asked if they had ever wanted to access the SSF but have not been able to. Response options were: yes, no, not sure. Those who selected “yes” were asked why (check all that apply: SSF was closed, line was too long, bad weather/outdoor location, concerns about COVID-19, other).

*COVID-19 variables*. A series of questions inquired about COVID-19-related changes in substance and service use. Reported changes in substance use since the start of COVID-19 included response options (yes/no) for: paid more for substances, used alone more often, could not find substance, used more of a substance, used a different substance, or changed dealer or source. Reported changes in harm reduction practices since the start of COVID-19 included response options (yes/no) for: used safe supply, used overdose prevention sites, used less, used more slowly, got drugs checked, did not share equipment, cleaned surfaces/supplies more often, carried naloxone, and stocked up on supplies. Changes in frequency of substance use compared to before COVID-19 was asked of participants, who selected from: increased; stayed about the same; or decreased. Changes in SSF usage compared to before COVID-19 were asked about (visiting more often/ visiting the same amount/visiting less often). Mental health changes compared to before COVID-19 included five options: much worse now; somewhat worse now; about the same; somewhat better now; and much better now. Changes in community connectedness compared to before COVID-19 was asked as “since COVID-19 began, have you felt any changes in how connected you feel to your community (however you define your own community)?” and included three options: stayed the same; more connected; and less connected. All variables included a “prefer not to say” option, and many included an “other” option with space for free text. For questionnaire items that listed multiple possible responses, participants selected all response options that applied to them.

### Analysis

To control for duplicate responses with anonymous questionnaire completion, we allowed participants to complete the questionnaire more than once but had a question on the survey to indicate whether someone had filled it out previously. Only the first completed questionnaire for each participant was retained for analysis. Responses of “prefer not to say” never totalled more than 3% of the sample and were dropped from bivariate and multivariate analyses. Bivariable and multivariable multinomial logistic regression were used to study factors associated with changes in frequency of SSF use and factors associated with SSF access issues. Sets of variables were tested in bivariable models and retained in the final multivariable model if they had a *p* value > 0.25. All analyses were completed using Stata 14.0.

### Community engagement

People with lived and living experience using drugs (“peer researchers”) and SSF site leadership team members were actively involved in the design and implementation of our study including involvement with developing the survey instrument, participating in data collection, data entry, analysis, interpretation of findings, and dissemination. The research team has been working closely on several joint research projects since 2017, shortly after the site opened, and together created a study to respond to the information needs of the SSF. Specifically, individuals in leadership roles at the SSF were involved in proposing the initial idea for the study, shaping the development of the funding proposal and co-executing the entire research project. Community engagement was facilitated through bi-weekly meetings with the study team, and capacity building exercises in data entry and data analysis. Through collaboration with an existing community-based research group of PWUD and harm reduction workers (called the Overdose Prevention Society Peer Research Assistant program, or “OPPRA”), the questionnaire was piloted before being used. This provided valuable information on the interpretability and acceptability of items and questionnaire length. The instrument was modified after piloting and returned to the OPPRA group for a secondary review. Data collection was jointly conducted by peer researchers and formally trained researchers. OPPRA members were offered paid data entry opportunities, where formally trained researchers created a database and showed interested OPPRA members how to transfer data from hard copy questionnaires into the database, and how to perform quality assurance checks on entered data. OPPRA members who were on the study team were also offered data analysis opportunities, where formally trained researchers gave step-by-step instructions on data cleaning and coding in Stata. Those interested in data analysis attended regular working meetings where we walked through univariate descriptive statistics and made coding decisions together, interpreted bivariable data, and decided which multivariate models to run.

Finally, local artists in the Downtown Eastside community were engaged in designing tailored posters that shared emerging findings from our questionnaire with SSF users (see Additional file 2: Appendix B). Large posters with study findings were brought to the SSF and put on display in the outdoor space for SSF users to engage with. Peer researchers stood with the posters and talked to SSF users about them, asking questions about how the study findings resonated with SSF users, what surprised them, and what next steps they wanted to see taken as a result of the findings. Smaller versions of the posters were displayed around the community, at other supervised consumption sites, community harm reduction sites, partner organizations, local social purpose businesses, and at street level in busy areas.

## Results

### Demographic and substance use characteristics

We received 200 returned questionnaires from participants, and after accounting for duplicate entries, the final sample of SSF users included 175 individuals of which 71% identified as men, 22% as women, and 5% as non-binary (2% did not record an answer). Demographic and substance use characteristics are given in Table 1. Most respondents (> 70%) were over 30 years old. In the month before filling out the questionnaire, most respondents were homeless. More specifically, this included 32% who slept on the street, 11% with friends/family, 11% at a shelter, and 9% in a tent. Most respondents indicated using heroin/fentanyl (85%) and/or crystal methamphetamine (66%) during their visit to the SSF, while all other substances were used by less than 20% of the sample (Table 1).

**Table 1:** Demographic and substance use characteristics of SSF users, Vancouver, Canada (2020), *n* = 175^

Characteristic	Total *n* (%)
*Demographics*	
Age	
20–29	46 (26.9)
30–39	66 (38.6)
40–49	32 (18.7)
50–59	20 (11.7)
≥ 60	5 (2.9)
Gender	
Man	121 (70.7)
Woman	37 (21.6)
Non-binary	9 (5.3)
Homeless (yes)^†^	97 (59.5)
Substances used on day of visit*	
Heroin/Fentanyl	149 (85.1)
Crystal methamphetamine	116 (66.3)
Cannabis	36 (20.6)
Alcohol	21 (12.0)
Crack	20 (11.4)
Cocaine	12 (6.9)
Other	12 (6.9)
Substance use frequency	
≥ Daily stimulant use^‡^	118 (73.8)
≥ Daily opioid use^‡^	150 (93.2)
Reported changes in personal substance use since COVID-19*	
Paid more for substances	97 (55.4)
Used alone more often	71 (43.7)
Changed dealer or source	68 (38.9)
Increased frequency	58 (40.9)
Could not find substance	57 (32.6)
Used different substance	35 (20.0)

^‡^Refers to the 14-day period prior to the baseline questionnaire

^†^refers to the month prior to the baseline questionnaire

^Analytic N may vary slightly due to item non-response

*Variables allowed for selection of multiple options

### Substance use frequency and changes to use

Over 90% of respondents indicated using opioids daily and 74% indicated using stimulants daily (Table 1). Respondents were asked about changes to substance use since the start of the COVID-19 pandemic. Forty-four per cent (44%) of respondents indicated using alone more often and over half (55%) also indicated that they now pay more for their substances than they did before the COVID-19 pandemic. At least one third of respondents also indicated that they had to change their source (39%) or had trouble finding substances (33%). Compared to before the start of COVID-19, 41% of respondents reported using substances more frequently at the time of participation.

### Service use and harm reduction practices

Respondents were asked about changes in their community connectedness, mental health, harm reduction practices, and SSF usage since the onset of the COVID-19 pandemic (responses in Table 2). Of note, 47% of the sample reported feeling less connected to their community now, with 23% reporting somewhat worse mental health, and 27% reporting much worse mental health since the start of COVID-19. Regarding changes to harm reduction practices, using the SSF was the most frequently selected option (Table 2), and respondents also reported a variety of harm reduction practices changes that they developed and implemented to keep themselves safe including not sharing equipment, accessing safer supply (defined as a “pharmaceutical-grade alternative to the toxic street supply” [27]), cleaning supplies and surfaces, and stocking up on harm reduction supplies. During the month before completing the questionnaire, 65% of respondents indicated accessing the SSF at least daily and 44% of respondents reported accessing the site more often since the onset of the pandemic (Table 2).

**Table 2:** Barriers to accessing SSF and changes to harm reduction practices with COVID-19 pandemic onset, Vancouver, Canada (2020), *n* = 175^

Characteristic	Total (*n*, %)
SSF usage	
≥ Daily SSF usage (past 14 days)	107 (65.2)
Reported SSF usage since COVID-19	
Increased	70 (44.6)
No change	61 (38.9)
Decreased	26 (16.6)
SSF access issues	
Ever had issues accessing SSF	116 (72.5)
Reason for SSF access issues*	
Wait too long	80 (45.7)
Closed	69 (39.4)
Poor weather	38 (21.7)
COVID-19 concerns	29 (16.6)
Reported harm reduction practice changes since COVID-19*	
Used SSF	68 (38.9)
Did not share equipment	67 (38.3)
Accessed safe supply	63 (36.0)
Cleaned supplies/surfaces	58 (33.1)
Stocked up on harm reduction supplies	58 (33.1)
Got drugs checked	55 (31.4)
Carried naloxone	48 (27.4)
Used more slowly	32 (18.3)
Used less drugs	27 (15.4)
Reported changes in mental health since COVID-19	
Much better	3 (1.9)
Somewhat better	11 (7.1)
About the same	63 (40.7)
Somewhat worse	42 (27.1)
Much worse	36 (23.2)
Reported changes in community connection since COVID-19	
Less connected	70 (47.3)
Stayed the same	57 (38.5)
More connected	21 (14.2)

^Analytic N may vary slightly due to item non-response

*variables allowed for selection of multiple options

### Factors associated with SSF use and access

The majority of study respondents (65%) used the site at least daily, and a smaller proportion (21%) several times per week, just once per week (6%) or less than once per week (8%). Many (73%) of these respondents reported experiencing issues accessing the site at some point. Overall, the most commonly reported barriers were having to wait too long (46%) followed by the site being closed (39%). We examined factors associated with having SSF access issues using bivariable logistic regression and did not find any significant associations between demographic and substance use characteristics and barriers to SSF access (results not shown).

We modelled factors associated with changes in SSF utilization since the start of the COVID-19 pandemic using multinomial logistic regression. From the bivariable models, represented in Table 3, respondents who reported experiencing access issues had 5.4 times the likelihood of decreased SSF utilization compared to those who did not; however, results did not remain significant following multivariable analysis (as shown in Table 4). In multivariable models, respondents who reported an increase in substance use had 7.7 times the likelihood of increased SSF usage compared to respondents whose substance use did not change. Mental health and community connectedness were not associated with changes in SSF utilization, nor were participant demographics, in either bivariable or multivariable models.

**Table 3:** Bivariable multinomial logistic regression of SSF usage since COVID-19, Vancouver, Canada (2020)

Characteristic	Model 1a: SSF usage since COVID-19 relative to no change:			
	A: Increased usage		B: Decreased usage	
	RRR	95% CI	RRR	95% CI
Demographics				
Age (/ age < 30)				
30–39	1.55	0.64–3.76	0.75	0.24–2.38
≥ 40	1.78	0.71–4.50	1.13	0.35–3.57
Homeless (/housed)^†^	1.18	0.57–2.41	1.31	0.50–3.45
Men (/women + non-binary)	0.62	0.27–1.41	0.49	0.17–1.41
SSF access issues, ever (/no)				
Yes	1.27	0.59–2.75	5.36*	1.14–25.2
Behavioural changes since COVID-19				
Substance use (/ no change)				
Increased	6.94**	2.93–16.44	2.10	0.63–6.96
Decreased	0.73	0.13–4.07	3.50	0.87–14.13
Used alone more often (/ no change)	1.99	0.90–4.40	2.71	0.87–8.40
Mental health since COVID-19				
Mental health (/ much worse)				
Somewhat worse	0.70	0.25–2.01	0.40	0.10–1.64
About the same	0.51	0.19–1.33	0.50	0.29–2.09
Community connection since COVID-19 (/ more connected)				
About the same	1.09	0.33–3.61	0.57	0.14–2.29
Less connected	1.08	0.33–3.49	0.59	0.15–2.28
Model statistic: *p* < 0.05				

Model is estimated with each comparison relative to no change in SSF usage as the reference group

*CI* confidence interval, *RRR* relative risk ratio

/ omitted reference category, *p < 0.05, **p < 0.001, Analytic significance level is set to *p* = 0.05, ^†^refers to the month prior to the baseline questionnaire

**Table 4:** Multivariable multinomial logistic regression of SSF usage since COVID-19, Vancouver, Canada (2020)

Characteristic	Model 1b: SSF usage since COVID-19 relative to no change:			
	A: Increased usage		B: Decreased usage	
	RRR	95% CI	RRR	95% CI
Demographics				
Age (/ age < 30)				
30–39	1.63	0.48–5.55	0.35	0.08–1.59
≥ 40	1.21	0.34–4.29	0.48	0.11–2.10
Homeless (/housed)^†^	1.49	0.53–3.80	1.67	0.44–6.29
Men (/women + non-binary)	0.32	0.10–1.08	0.29	0.07–1.25
Behavioural changes since COVID-19				
Used alone more often (/ no change)	1.47	0.57–3.81	2.46	0.71–8.49
Substance use (/ no change)				
Increased	7.70	2.73–21.75	1.58	0.36–6.81
Decreased	0.95	0.08–10.96	4.22	0.59–30.0
Model statistic: *p* < 0.05				

Model is estimated with each comparison relative to no change in SSF usage as the reference group

*CI* confidence interval, *RRR* relative risk ratio

/ = omitted reference category, *p < 0.05; **p < 0.001, Analytic significance level is set to *p* = 0.05, ^†^refers to the month prior to the baseline questionnaire

On free-text questions that asked what future sites should learn or copy from the SSF, the peer-led model was cited as an integral part of the SSF operation, with many respondents stressing the importance of having staff who understand the lived and living experience of people who use drugs. When asked what future sites should do differently from the current SSF, respondents highlighted the importance of having better shelter from wind and rain, heaters, access to food/water, and the desire for an indoor inhalation space.

## Discussion

We surveyed clients accessing harm reduction services at an outdoor SSF in Vancouver, Canada, to characterize client demographic and substance use behaviours, and to document harm reduction and service utilization practices among clients following the onset of the COVID-19 pandemic. Most respondents identified as men, were homeless or housing insecure, and were over 30 years old, similar to the clients served by other supervised consumption sites permitting inhalation worldwide [6]. These are all characteristics about SSF users in this context that were previously unknown. Most respondents reported they were using opioids and/or crystal methamphetamine on the day of their visit to the SSF, and almost all respondents reported daily use of one or both types of substances, indicating that those who use the SSF were using drugs frequently. These findings also signal that a high proportion of SSF clients engage in polysubstance use, a risk factor for overdose and an indicator of service need [28, 29]. This finding is echoed by recent coroner’s reports showing an increased prevalence of stimulants in opioid overdoses [7, 24], and is aligned with an observed trend of initiation of methamphetamine use by people who use opioids [30] and increases in methamphetamine-related emergency room admissions [31].

We also documented previously unknown changes in substance use and harm reduction behaviours that have been adopted since the start of COVID-19 for SSF users. More than half of respondents had to pay more for their substances, a substantial hardship given the high proportion of respondents who were homeless and the large proportion with daily use. Respondents adopted a variety of harm reduction practices with the onset of COVID-19, speaking to the innovativeness and ability of PWUD to respond to increasingly harmful and rapidly changing drug use environments, even in times when government responses or public guidance is slow, inadequate, unavailable or otherwise highly constrained. Like other harm reduction services facing similar challenges during the pandemic [22], adaptations for the SSF were undertaken with considerations of safety among PWUD. A central focus on peer-led service delivery differentiates this SSF from others globally [6] and many of the rapid and necessary COVID-19 site changes were implemented by peer workers at the SSF who had direct experience in meeting the needs of PWUD.

Although many respondents had at one time experienced issues in accessing the SSF (most commonly due to operating hours or having to wait to get in), there were no significant associations between variables we measured and SSF access issues. Here, we note that the absence of significant associations between demographic and substance use characteristics and experiencing SSF access issues may be a positive finding from an equity perspective: there are no factors that we found that increased risk of having access challenges, even though many participants experienced them. This is likely related to the demand for services far outpacing the resources and capacity to respond to that need, and points to the urgent need for more investment in SSF sites like the one we studied. Notably, the site did not decrease operating hours during the COVID-19 pandemic, so wait time mentioned by clients were due to increased demand for the site, rather than site changes. Relative to other SSFs globally [6], there are very few eligibility criteria, rules, or restrictions on service at the SSF in which we conducted our study, possibly contributing to reduced access barriers for clients of the SSF. However, just because we did not identify demographic barriers to access does not mean they do not exist at the SSF. Other research on supervised consumption sites has identified gender-related barriers to access of such services [14], and we may have been unable to detect these issues given our sample size and methodological approach. In particular, there may be important barriers for pregnant and parenting people and racialized and minoritized populations at supervised consumption facilities and this is something that should be explored in future research.

Many respondents reported a notable increase in substance use frequency since the onset of the COVID-19 pandemic. For some, this also meant using drugs alone more often: a phenomenon also seen in other recent studies and a concern given documented linkages with increased risk of overdose fatality [32]. This points to the need for expansion of services to support PWUD such as overdose detection devices, more supervised consumption facilities, longer operating hours and more funding for existing sites, and increased access to lifesaving overdose reversal medication. For other respondents, this increased use was associated with an increase in SSF utilization. Importantly, increased drug use was the only variable associated with changes in SSF utilization and this speaks to a direct correlation between increased need for services and increased service utilization, which again points to a need for investment in SSF sites. Given the prevalence and frequency of polysubstance use, vulnerable housing situations and severity of substance use disorder a majority of SSF users in our study face, the importance of the life-saving services provided by SSFs and the integral role SSFs play in the continuum of harm reduction services cannot be overstated. Still, implementation remains slow and insufficient, possibly due to the high cost associated with retrofitting buildings to make indoor smoking facilities that meet local health and safety guidelines, and the insufficient political will to fund the necessary retrofits.

### Limitations

This exploratory and community-based study was not intended to provide representative or epidemiological data, limiting the generalizability of our findings. We relied on self-reported data, which is vulnerable to a variety of reporting biases, though, this common concern with research among PWUD has been met with multiple reports of high reliability [33–36]. Given our study purpose, our data collection constraints, and our privacy considerations, we collected limited demographic information (and specifically no race/ethnicity data) from respondents and therefore may not have captured all the factors that could be associated with access barriers or SSF utilization frequency. Further, because we sampled from SSF users who were at the site, the perspectives and practices of those experiencing access barriers may not be well-represented in our study. Specifically, those already accessing the SSF overcame their access barriers and there may be people who did not access the site at all due to barriers, which would not be captured in our study. The findings presented in this paper should not be considered representative of SSF users or PWUD more broadly. Rather, we reached a critical sub-population of PWUD who utilize a rare but vitally important service, centring our work on the SSF’s information needs, in a space in which there is extremely limited data.

## Conclusions

This study represents one of very few sources of published information about SSF users in North America, barriers to accessing SSFs, and current harm reduction and drug use practices. Future research is needed to better understand how to address the barriers for SSF users that were identified in our study to support the scale up of SSF sites globally. As we continue to live with the catastrophic impact of drug poisoning and overdose, we will need to identify how SSFs can expand and adapt their services to meet the needs of those who are underserved due to their preferred route of administration and at high risk of overdose. Funding for SSFs and implementation of SSFs is critically needed to support these service expansions.

## Supplementary Information

**Additional file 1.** **Appendix A.** Inhalation Tent Survey.

**Additional file 2.** **Appendix B.** Posters created to facilitate community engagement and shared interpretation of study findings.

## Data Availability

The datasets used and/or analysed during the current study are available from the corresponding author on reasonable request.

## Abbreviations

SSF: Supervised smoking facility
PWUD: People who use drugs
COVID-19: Coronavirus disease of 2019
