# Supplementary material for: Supervised smoking facility access, harm reduction practices, and substance use changes during the COVID-19 pandemic: a community-engaged cross-sectional study
Source: Harm Reduct J. 2023 Jul 31;20:101. doi: 10.1186/s12954-023-00825-7 (PMC10388471; doi:10.1186/s12954-023-00825-7)
Supplement: Supplementary file 1 — Additional file 1. Appendix A. Inhalation Tent Survey. [file 12954_2023_825_MOESM1_ESM.docx]

**1.** **Have you completed this survey before?**

( ) Yes ( ) No

( ) Not sure ( ) Prefer not to say

***About yourself…***

**2. What is your gender?**

( ) Man ( ) Woman

( ) Non-binary

( ) Another category:__________________

( ) Prefer not to say

**3. What is your age?**

( ) Under 19 ( ) 20 – 29

( ) 30 - 39 ( ) 40 – 49

( ) 50 – 59 ( ) 60+

( ) Prefer not to say

**4. Where did you sleep most nights in the last month?**

( ) Street ( ) Tent

( ) Shelter ( ) Hotel

( ) Staying with family or friends

( ) Your own house or apartment

( ) Prefer not to say

( ) Other:_______________________

***About substance use…***

**5. What substances are you using at the inhalation tent today? [Check all**

**that apply]**

( ) Crystal meth ( ) Crack

( ) Heroin/fentanyl ( ) Cocaine

( ) Cannabis ( ) Alcohol

( ) Other:____________________

( ) I’m not using substances

( ) Prefer not to say

**7. How often did you use the OPS Inhalation Tent in the past 14 days?**

( ) More than once per day

( ) Every day

( ) Several times per week

( ) Once per week

( ) Less than once per week

( ) Never ( ) Prefer not to say

**9. How often did you use stimulant drugs (e.g., crystal meth, crack, cocaine) in the past 14 days?**

( ) More than once per day

( ) Every day

( ) More than once per week

( ) Once per week

( ) Less than once per week

( ) Never ( ) Prefer not to say

**10.** **How often did you use heroin, fentanyl or down in the past 14 days?**

( ) More than once per day

( ) Every day

( ) More than once per week

( ) Once per week

( ) Less than once per week

( ) Never ( ) Prefer not to say

**11. Have you ever wanted to access the OPS Inhalation Tent but have not been able to?**

( ) Yes ( ) No

( ) Not sure ( ) Prefer not to say

**12. If yes, why [Check all that apply]?**

( ) Inhalation tent was closed (wrong hours)

( ) Line was too long

( ) Bad weather / was outdoors

( ) Concerns about COVID-19

( ) Much better now ( ) Prefer not to say

Other:___________________________

***About COVID-19…***

**13. Compared to before COVID-19, has the frequency of your substance use changed?**

( ) Increased since the start of COVID-19

( ) Stayed about the same

( ) Decreased since the start of COVID-19

( ) I don’t use substances

( ) Other:______________________________

( ) Prefer not to say

**14. Compared to before COVID-19, have you visited the OPS Inhalation Tent more often?**

( ) Yes, I visit more often since COVID-19

( ) I visit the Inhalation tent the same amount

( ) No, I visit less often since COVID-19

( ) Other:______________________________

( ) Prefer not to say

**15. Are you satisfied with the safety measures the OPS Inhalation Tent has put in place to prevent the spread of COVID-19?**

( ) Yes, I am satisfied

( ) No, the inhalation tent should do more to prevent the spread of COVID-19.

(describe: __________________________

___________________________________)

( ) Not sure ( ) Prefer not to say

**16. Have you experienced any of the following changes to your substance use since COVID-19**

**[Check all that apply]?**

( ) Paid more for your substances

( ) Changed your source or dealer

( ) Unable to find the substance you were seeking

( ) Used a different substance than you were seeking

( ) Other:_____________________________

( ) Prefer not to say

**17. Since COVID-19, have you made any of the following changes to your harm reduction practices [Check all that apply]?**

( ) Use safe supply

( ) Get drugs checked

( ) Use overdose prevention sites

( ) Use less

( ) Use more slowly

( ) Don’t share equipment

( ) Clean surfaces/supplies more often

( ) Carry naloxone

( ) Stock up on supplies

( ) Other: _________________________

( ) Prefer not to say

**18. Have you used substances alone more often since COVID-19?**

( ) Yes, I use substances alone more often

**THANK YOU FOR COMPLETING THIS QUESTIONNAIRE!!** Submitting this questionnaire means you consent to your data being used for research and you may not withdraw your consent later, because the surveys are anonymous. If you have any questions about this research you can talk to xxx.

( ) No, I use substances alone the same amount

( ) No, I never use substances alone

( ) Other:______________________________

( ) Prefer not to say

**19. Since COVID-19, how has your mental health changed?**

( ) Much worse now

( ) Somewhat worse now

( ) About the same

( ) Somewhat better now

( ) Much better now ( ) Prefer not to say

Other:___________________________

**20. Since COVID-19 began, have you felt any changes in how connected you feel to your community (however you define your own community)?**

( ) Stayed the same ( ) More connected

( ) Less connected ( ) Prefer not to say

( ) Other:___________________________

***Future Inhalation Site suggestions…***

**21. What changes would you make if you were to design a new safe consumption space for smoking substances [Check all that apply]?**

( ) Washroom and handwashing facilities

( ) Indoor location

( ) Area to relax or socialize (e.g., chill zone)

( ) Integration with health and social services

( ) Guidance on how to use substances

( ) Fewer crowds

( ) Access to COVID-19 testing

( ) Easier access to masks, gloves, or other personal protective equipment

( ) Other: _________________________

( ) Prefer not to say

**22. Some organizations in Canada want to open new Inhalation Tents. What you would recommend they copy from the OPS Inhalation Tent model?** ______________________________________________________________________________

**What would you recommend they do differently?**____________________________________________________________________

**23. Is there anything else you want to add?**

_______________________________________
