# Supplementary material for: Supervised smoking facility access, harm reduction practices, and substance use changes during the COVID-19 pandemic: a community-engaged cross-sectional study
Source: Harm Reduct J. 2023 Jul 31;20:101. doi: 10.1186/s12954-023-00825-7 (PMC10388471; doi:10.1186/s12954-023-00825-7)

Appendix A. Posters created to facilitate community engagement and shared interpretation of study findings


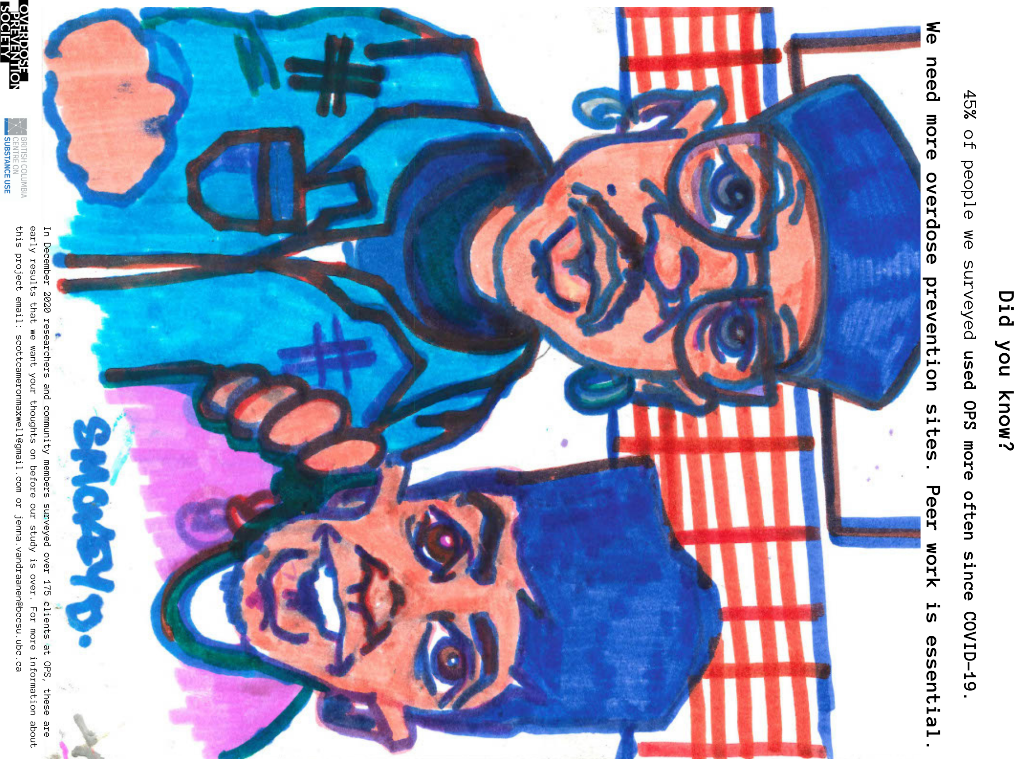


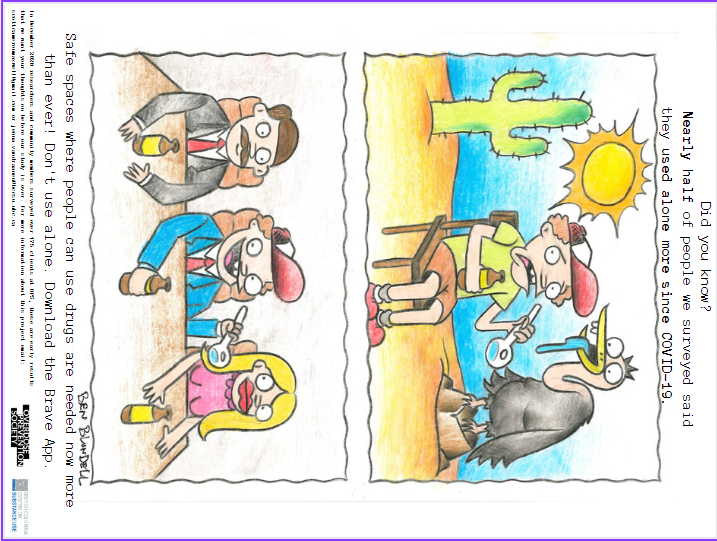


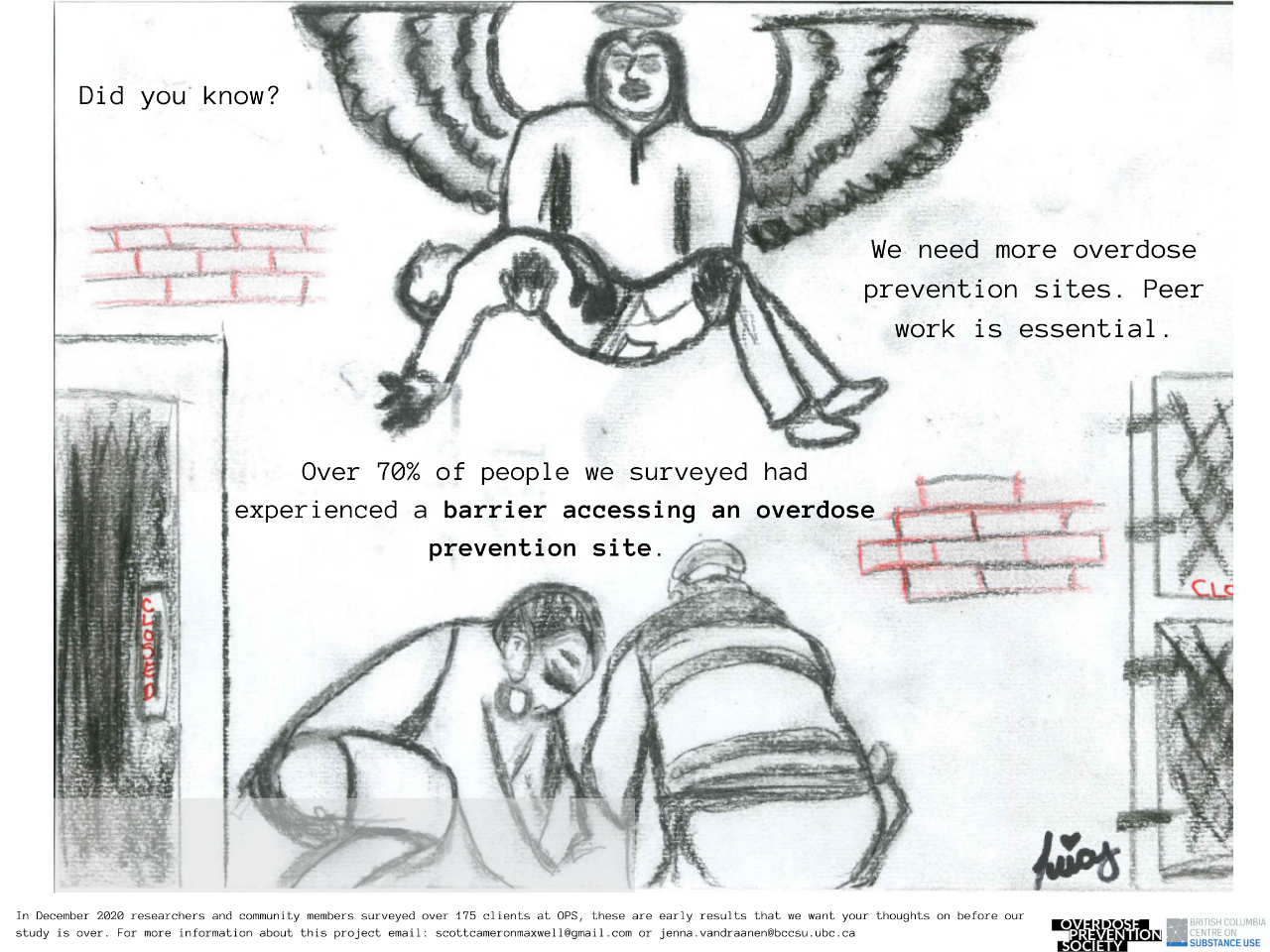


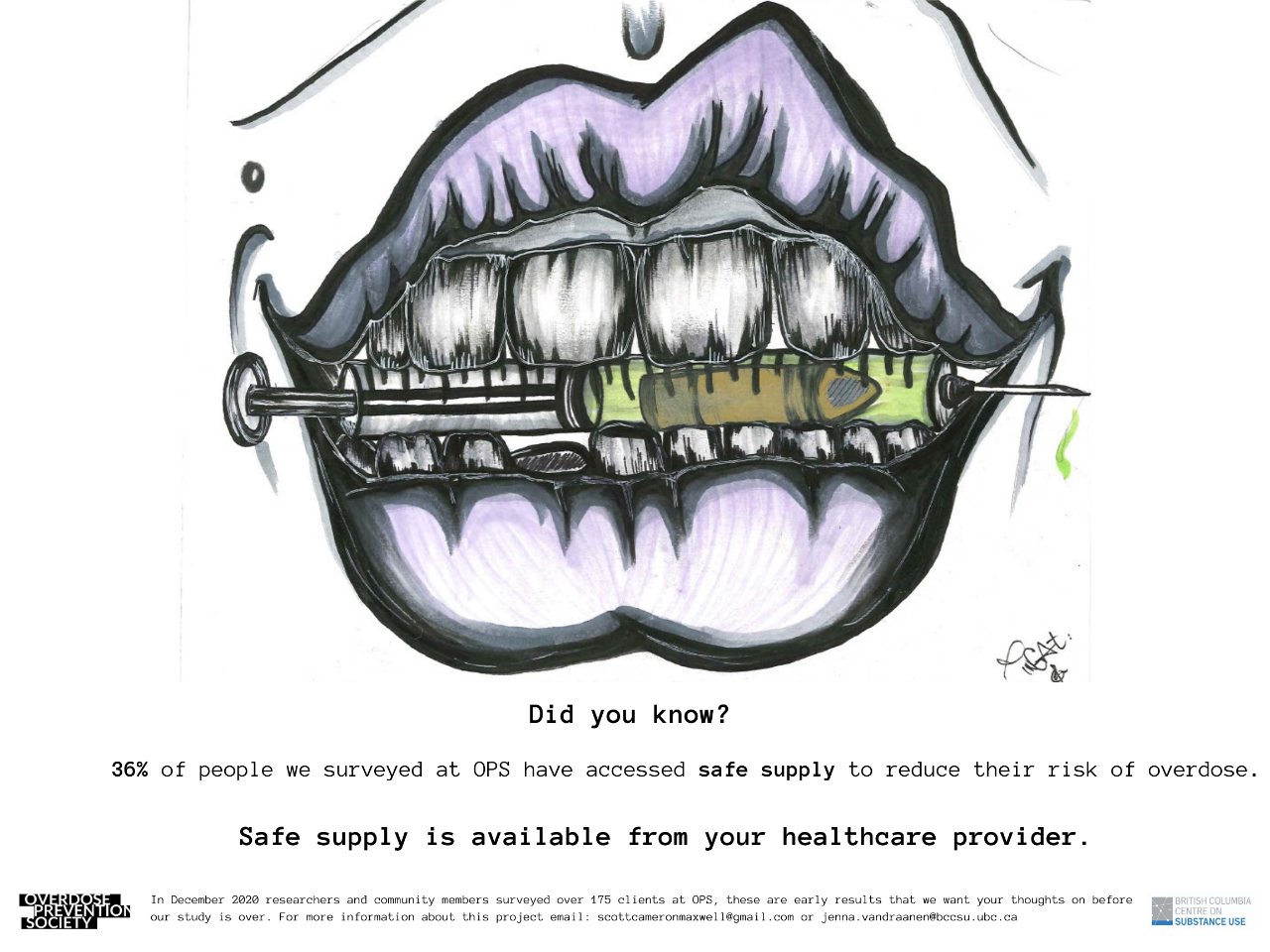

Supplement: Supplementary file 2 — Additional file 2. Appendix B. Posters created to facilitate community engagement and shared interpretation of study findings. [file 12954_2023_825_MOESM2_ESM.docx]
